# Does atenolol differ from other β-adrenergic blockers?

**DOI:** 10.1186/1472-6904-7-4

**Published:** 2007-05-08

**Authors:** Ivar Aursnes, Jan-Bjørn Osnes, Ingunn Fride Tvete, Jørund Gåsemyr, Bent Natvig

**Affiliations:** 1 Department of Pharmacotherapeutics, University of Oslo, 0316 Blindern, Oslo, Norway; 2 Department of Pharmacology, University of Oslo, 0316 Blindern, Oslo, Norway; 3 Department of Mathematics, University of Oslo, 0316 Blindern, Oslo, Norway

## Abstract

**Background:**

A recent meta-analysis of drug effects in patients with hypertension claims that all β-adrenergic blockers are equally effective but less so than other antihypertensive drugs. Published comparisons of the β-adrenergic blocker atenolol and non-atenolol β-adrenergic blockers indicate different effects on death rates, arrhythmias, peripheral vascular resistance and prognosis post myocardial infarction, all in disfavour of atenolol. In keeping with these findings, the data presented in the meta-analysis indicate that atenolol is less effective than the non-atenolol β-adrenergic blockers both when compared with placebo and with other antihypertensive drugs. These findings were not, however, statistically significant.

**Methods:**

We performed an additional analysis with a Bayesian statistical method in order to make further use of the published data.

**Results:**

Our calculations on the clinical data in the meta-analysis showed 13% lower risk (risk ratio 0.87) of myocardial infarction among hypertensive patients taking non-atenolol β-adrenergic blockers than among hypertensive patients taking atenolol. The 90 % credibility interval ranged from 0.75 to 0.99, thereby indicating statistical significance. The probability of at least 10% lower risk (risk ratio ≤ 0.90), which could be considered to be of clinical interest, was 0.69.

**Conclusion:**

Taken together with the other observations of differences in effects, we conclude that the claim that all β-adrenergic blockers are inferior drugs for hypertensive patients should be rejected. Atenolol is not representative of the β-adrenergic blocker class of drugs as a whole and is thus not a suitable drug for comparisons with other antihypertensive drugs in terms of effect. The non-atenolol β-adrenergic blockers should thus continue to be fundamental in antihypertensive drug treatments.

## Background

In a recent meta-analysis, Lindholm, Carlberg and Samuelsson claim that, in patients with hypertension, the β-adrenergic blocker atenolol is no different from other β-adrenergic blockers in terms of effects on hard endpoints [1]. This assertion brings to mind the HAPPHY trial [2] in which patients on atenolol showed higher death rates and patients on metoprolol lower death rates than did patients taking diuretics. The difference between the two outcomes was not statistically significant. For other endpoints, only pooled results were released from that study.

### Animal studies

Atenolol is water-soluble and is therefore distributed to the brain to a much lower extent than most other β-adrenergic blockers, which are lipid-soluble. It has been hypothesized that central nervous stimulation of the vagal tone by β-adrenergic blockers counteracts a liability towards ventricular fibrillation. In this connection, water-soluble atenolol is far less effective than a lipid-soluble β-adrenergic blocker such as metoprolol. Unfortunately, this contention has been studied only in dogs and published only in abstract form [3].

### Pathophysiology

Somewhat surprisingly, long-term (> 6 months) exposure of hypertensive patients to β-adrenergic blockers results in peripheral vasodilatation. This occurs with metoprolol and other β-adrenergic blockers, as well as with other drugs, but not with atenolol [4]. A corresponding difference was found between atenolol and the various other drugs as regards the media to lumen ratios of small arteries.

### Post myocardial infarction

Unlike the other β-blockers, atenolol has not been shown to improve the long-term prognosis after myocardial infarction [5], nor has it been tested and shown to be an effective treatment for heart failure.

In light of the above we examined the summaries of the data in the published meta-analysis [1]. The authors found that the risk ratio for occurrence of myocardial infarction in hypertensive patients was (with 95 % confidence intervals) 0.86 (0.67 – 1.11) for non-atenolol β-blockers compared with other antihypertensive drugs but for atenolol versus other antihypertensive drugs the risk ratio was 1.05 (0.91 – 1.21). This strongly indicates a difference between non-atenolol β-blockers and atenolol. Moreover, a similar difference was found when non-atenolol β-blockers and atenolol were compared with placebo, with a risk ratio of 0.89 (0.74 – 1.06), i.e. decreased risk for the former but a risk ratio of 0.99 (0.83 – 1.19), i.e. no decreased risk for atenolol.

We undertook the task of finding numerical expressions for the suspicion of a difference between atenolol and non-atenolol β-blockers by using the data available in the meta-analysis [1].

## Methods

We combined the information provided by the two sets of data, thereby obtaining stronger statistical power. Eight of the studies in the analysis compared atenolol and non-atenolol β-blockers, respectively, with other antihypertensive drugs and six compared atenolol and non-atenolol β-blockers, respectively, with placebo. We used a previously described Bayesian procedure [6] and first combined the results of studies in similar treatment groups, assuming that the relative effects of the treatment in the various studies were comparable, even though the absolute risks might differ. This is a natural assumption to make in this context as the basis of the Bayesian analysis in which results from different studies are combined. Generally, it is difficult to verify the validity of this assumption against data. However, from the data in the published meta-analysis [1] the assumption seems reasonable, except for the MRC Old comparison of atenolol with other antihypertensive drugs. Hence, calculations were also carried through without this comparison.

We then calculated the relative risk of non-atenolol β-blockers against atenolol by dividing the corresponding risk ratios. Finally, based on the 8 and 6 studies respectively, we combined the two estimates of the risk ratios for myocardial infarction in patients treated with non-atenolol β-blockers versus atenolol by simply updating one with the other by means of Bayes' formula.

## Results

Table 1 shows our findings, indicating that the frequency of myocardial infarctions is 13 % lower in patients on a non-atenolol β-blocker compared with patients on atenolol based on all 14 studies. This outcome was to be expected, since it agrees with previously published observations [2], which provide justification for relying on the 90 % credibility interval. This interval 0.750 – 0.992 has an upper border slightly less than unity. The 95 % credibility interval was 0.727 – 1.023. The probability of at least 10% lower risk (risk ratio ≤ 0.90), which could be considered to be of clinical interest, was 0.689. The results are slightly weaker when the MRC Old study is excluded (Table 1).

**Table 1:** Risk ratios for myocardial infarction among hypertensive patients treated with non-atenolol versus atenolol β-blockers

	Number of studies	Mean Risk ratio	Median risk ratio	Credibility interval		Probability of risk ratio ≤ 0.90
				2.5 – 97.5 %	5 – 95 %	
Studies that only included comparison with non-β-blockers	8	0.836	0.828	0.633 – 1.078	0.664 – 1.031	0.836
Studies that only included comparison with placebo	6	0.918	0.906	0.711 – 1.172	0.734 – 1.125	0.475
All studies included	14	0.868	0.867	0.727 – 1.023	0.750 – 0.992	0.689
All studies except MRC Old atenolol versus other antihypertensive drugs	13	0.882	0.883	0.742 – 1.039	0.758 – 1.016	0.616

Four outputs with 100 000 iterations each of Monte Carlo sampling with a Bayesian method giving ratios of risks of myocardial infarctions in hypertensive patients using non-atenolol β-adrenergic blockers versus using atenolol, respectively, with data from studies involving non β-blocker drugs, studies with placebo groups and with data from both kinds of studies

## Discussion

The present additional statistical analysis of the published data [1] thus shows a different outcome for atenolol in relation to the non-atenolol β-blockers, in disfavour of atenolol based on all 14 studies. Our finding also agrees with statements by Carlberg, Samuelsson and Lindholm in their earlier meta-analysis [7]. One of the implications of this different outcome is that atenolol is not representative for the β-adrenergic blocker class of drugs as a whole. Observations relating to atenolol do not necessarily apply to other β-adrenergic blockers. A similar reservation has also been made recently by others [8]. Therefore we do not agree with an editorial on the publication of the ASCOT trial and the meta-analysis, which advocates that the current guidelines for the treatment of hypertension should be changed in disfavour of all β-adrenergic blockers [9].

## Conclusion

Claims have been made that β-adrenergic blockers are inferior drugs when used to treat patients with hypertension. We suggest that a recent meta-analysis shows that these claims are correct only in the case of atenolol. We have substantiated this suggestion by re-analysing the published data using a Bayesian technique. This re-analysis showed 13 % fewer myocardial infarctions among patients treated with non-atenolol β-adrenergic blockers than among patients treated with atenolol. We argue that non-atenolol β-adrenergic blockers should continue to be fundamental in the treatment of hypertension.

## Competing interests

The authors declare that they have no competing interests except that JBO has received lecture fees from 'Orion' and 'Merck'.

## Authors' contributions

IA and JBO, presented the problem and drafted the manuscript along with BN, who suggested the statistical solution based on earlier work by some of the present authors [6]; IFT did the computations and took part in the planning along with JG. All authors read and approved the final manuscript.

## Pre-publication history

The pre-publication history for this paper can be accessed here:


